# Combined treatment with N‐acetylcysteine and gefitinib overcomes drug resistance to gefitinib in NSCLC cell line

**DOI:** 10.1002/cam4.2610

**Published:** 2019-12-31

**Authors:** Jun Li, Xiao‐Hui Wang, Jing Hu, Meng Shi, Lu Zhang, Hong Chen

**Affiliations:** ^1^ Department of Pulmonary and Critical Care Medicine The First Affiliated Hospital of Chongqing Medical University Chongqing China

**Keywords:** epithelial‐mesenchymal transition, gefitinib resistance, N‐acetylcysteine, NSCLC, Src

## Abstract

We aimed to explore the molecular substrate underlying EGFR‐TKI resistance and investigate the effects of N‐acetylcysteine (NAC) on reversing EGFR‐TKI resistance. In the current research, the effects of NAC in combination with gefitinib on reversing gefitinib resistance were examined using CCK‐8 assay, combination index (CI) method, matrigel invasion assay, wound‐healing assay, flow cytometry, western blot, and quantitative real‐time PCR (qRT‐PCR). CCK8 assay showed that NAC plus gefitinib combination overcame EGFR‐TKI resistance in non‐small cell lung cancer (NSCLC) cells by lowering the value of half maximal inhibitory concentration (IC50). CI calculations demonstrated a synergistic effect between the two drugs (CI < 1). Matrigel invasion assay and wound healing assay demonstrated a decrease in migration and invasion ability of PC‐9/GR cells after NAC and gefitinib treatment. Flow cytometry displayed enhanced apoptosis in the combination group. Western blot and qRT‐PCR revealed that increased E‐cadherin and decreased vimentin in the combination group. When PP2 was administered with gefitinib, the same effects were seen. Our findings suggest that NAC could restore the sensitivity of gefitinib‐resistant NSCLC cells to gefitinib via suppressing Src activation and reversing epithelial‐mesenchymal transition.

## Graphical abstract

Our results demonstrated that NAC has synergistic effect to restore gefitinib sensitivity in gefitinib‐resistant NSCLC cells. These two drugs used in combination could overcome the resistance of PC‐9/GR to gefitinib by inhibiting Src activation and reversing EMT.

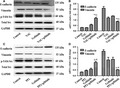

## 1 INTRODUCTION

Lung cancer, one of the most aggressive cancers, accounts for the leading cause of cancer‐related mortality worldwide,^1^, ^2^ and non‐small cell lung cancer (NSCLC) contributes to around 85% of lung cancer. Gefitinib and erlotinib, two major epithelial growth factor receptor tyrosine kinase inhibitors (EGFR‐TKIs), display promising therapeutic efficacy in patients with NSCLC carrying EGFR‐activating mutations (for example, exon 19 deletion and exon 21 L858R), and these patients regularly receive EGFR‐TKIs as a first‐line treatment.^3^, ^4^, ^5^, ^6^, ^7^ However, almost all patients become resistant to gefitinib and erlotinib within a median time period of approximately 10 months.^8^, ^9^, ^10^ However, the mechanisms of EGFR‐TKI resistance are largely unknown. Therefore, exploring the underlying mechanisms is beneficial for developing new strategies to overcome this problem, which might improve the prognosis of patients with NSCLC.

Currently, multiple factors are involved in the resistance mechanisms, including the T790M secondary mutation in EGFR, HGF overexpression, MET amplification, transition into small cell lung carcinoma, obtaining cancer stem‐cell phenotypes and epithelial‐mesenchymal transition (EMT).^11^, ^12^, ^13^, ^14^ EMT is a universal phenomenon in various physiological and pathological processes. It is clear that epithelial cells display characteristics of mesenchymal cells, accompanied by upregulation of vimentin and N‐cadherin, and downregulation of E‐cadherin during EMT. EMT also contributes to tumor invasion, proliferation, metastasis, and therapy resistance to EGFR‐TKIs.^15^, ^16^ As a consequence, targeting EMT might be a potential strategy to reverse or prevent EGFR‐TKIs resistance.

N‐acetylcysteine (NAC) is an effective antioxidant widely used in anticancer investigation in recent years. Our previous studies have demonstrated that NAC could overcome gefitinib resistance mediated by cigarette smoke extract (CSE). However, whether NAC plays a critical role in non‐smokers would explore the combined effect of NAC with gefitinib on gefitinib‐resistant cells and the underlying mechanisms.

## 2 MATERIALS AND METHODS

### 2.1 Cell culture

PC‐9 gefitinib‐sensitive cells (PC‐9)^17^, ^18^ and gefitinib‐resistant cells (PC‐9/GR) were gifts from Dr Jian Zhang at Xijing Hospital, Fourth Military Medical University, China. It is known that exon19 deletion is one of the hall‐marks in EGFR‐activating mutation, and PC‐9 cell line is characterized by exon19 deletion of lung cancer cells. The mutation profile is exon19(E746‐A750)del for PC‐9/GR.^19^ Cells were cultured in RPMI 1640 medium (Hyclone, USA) with 10% fetal bovine serum (PAN, USA) at 37°C in a cell incubator containing 5% CO_2_. In addition, PC‐9/GR cell was cultured in medium containing 10 nmol/L of gefitinib to maintain resistance.

### 2.2 Reagents

NAC, 4ʹ,6‐diamidino‐2‐phenylindole (DAPI), dimethyl sulfoxide (DMSO), and Triton X‐100 were purchased from Sigma‐Aldrich. PP2 and gefitinib were purchased from Tocris Bioscience and Abcam, respectively. Rabbit monoclonal antibodies against E‐cadherin (24E10), Src (32G6), Phospho‐Src (Ty416), and mouse monoclonal antibody against GAPDH were purchased from Cell Signaling Technology. Bax (WL01637) and Bcl‐2 (WL01556) were purchased from Wanleibio. Rabbit monoclonal antibody against Vimentin (EGFR3776) was purchased from Abcam.

### 2.3 Cell growth assay

Cell proliferation was evaluated with a CCK8 kit (Dojindo Laboratories). In brief, Cells (5 × 10^3^ cells) were seeded into 96‐well plates, cultured overnight and treated with various concentrations of drugs for 48 hours. Then, 10 µL of CCK8 was added to each well and cells were incubated for 2 hours. Optical density (OD) was set at 450 nm by Mircroplate Reader.

### 2.4 Combination studies

Combination studies were performed as described previously.^20^, ^21^ On the basis of the median‐effect analysis by Chou and Talalay (CalcuSyn software, Biosoft: Chou, 2010), the effects of drugs were calculated using the CI method for each experimental condition.^22^, ^23^

### 2.5 Western blotting analysis

The precise procedure was in accordance with our previous methods.^24^ In brief, proteins were separated by SDS‐PAGE gel and transferred to PVDF membranes. After blocking with 5% fat‐free milk or BSA, the membranes were incubated with primary antibodies overnight at 4°C, followed by incubation with secondary antibodies for 2 hours at room temperature. The protein bands were detected.

### 2.6 RNA isolation and qRT‐PCR

The procedure of RNA extraction and cDNA synthesis was based on our previous study.^25^ The following primers were used: E‐cadherin, 5‐CGTAGCAGTGACGAATGTGG‐3(F) and 5‐CTGGGCAGTGTAGGATGTGA‐3(R); vimentin, 5‐GAGTCCACTGAGTACCGGAG‐3(F) and 5‐ACGAGCCATTTCCTCCTTCA‐3(R); GAPDH, 5‐ACCTGACCTGTCTAGAA‐3(F) and 5‐TCCACCACCTGTTGCTGTA‐3(R). The relative expression of indicated mRNAs was normalized to GAPDH.

### 2.7 Cell invasion assay

Sixty microliters of Matrigel (Becton Dickinson) was added into the center of each chamber (Millipore). The cells were seeded in the upper chamber of the insert with or without drugs, after incubation for 24 hours at 37°C with 5% CO_2_. The upper surface of the insert was scraped and the cells on the lower surface of the membrane were fixed with methanol and stained with 0.1% crystal violet solution (Becton Dickinson). Cells in the bottom of the membrane were counted using a light microscope.

### 2.8 Measurement of cell migration

Cells were planted into six‐well plates to create a confluent monolayer with up to 80% cell confluence, and then starved for 24 hours with serum‐free medium, followed by scratching with a sterile 200 µL tip to manually create a wound. The cells were washed with PBS and cultured in medium supplemented with NAC, gefitinib or a combination of both. Images were acquired by inverted optical microscope after creating the wound.

### 2.9 Measurement of apoptotic rate

Apoptotic cells were determined by Annexin V‐FITC/propidium iodide (PI) Kit according to manufacturer's guidelines. The specific process referred to our previous work.^24^ Briefly, PC‐9/GR cells were treated with NAC, gefitinib or a combination of both for 48 hours, respectively. Afterward, cells were harvested, and stained with Annexin V‐FITC and PI according to manufacturer's recommendations (Beyotime Institute of Biotechnology).

### 2.10 Statistical analysis

The data were represented as mean ± standard deviation (SD) or 95% confidence interval (CI) for three independent experiments. The GraphPad Prism software (version 5.0, GraphPad Software) was used for statistical analysis. The comparison between two independent treatment groups was analyzed by unpaired, two‐tailed Student's *t* test. One‐way analysis of variance (ANOVA) was utilized to analyse the variance among multi‐sample. Statistical significance was assumed at *P* < .05.

## 3 RESULTS

### 3.1 Determination of dose‐response curves and PC‐9/GR EMT phenotype characteristics

Similar to our previous findings, the IC50 value of PC‐9/GR for gefitinib was 7.711 μmol/L (95% CI: 7.058‐9.657 μmol/L; Figure S1A) which was increased 141‐fold compared with that of PC‐9 cells (IC50: 0.05471 μmol/L, 95% CI: 0.04378‐0.06835 μmol/L; Figure S1B). This result showed a highly resistant effect to gefitinib in PC‐9/GR cells. As presented in Figure S1C, E‐cadherin was expressed in PC‐9 cells, but downregulated in PC‐9/GR cells. In addition, vimentin was upregulated in PC‐9/GR cells, which was absent in PC‐9 cells. These results demonstrated an EMT phenotype characteristic of PC‐9/GR cells. The IC50 of NAC in PC‐9/GR cells was 15.53 mmol/L (95% CI: 14.50‐16.62 mmol/L; Figure S1D).

**Figure 1:**
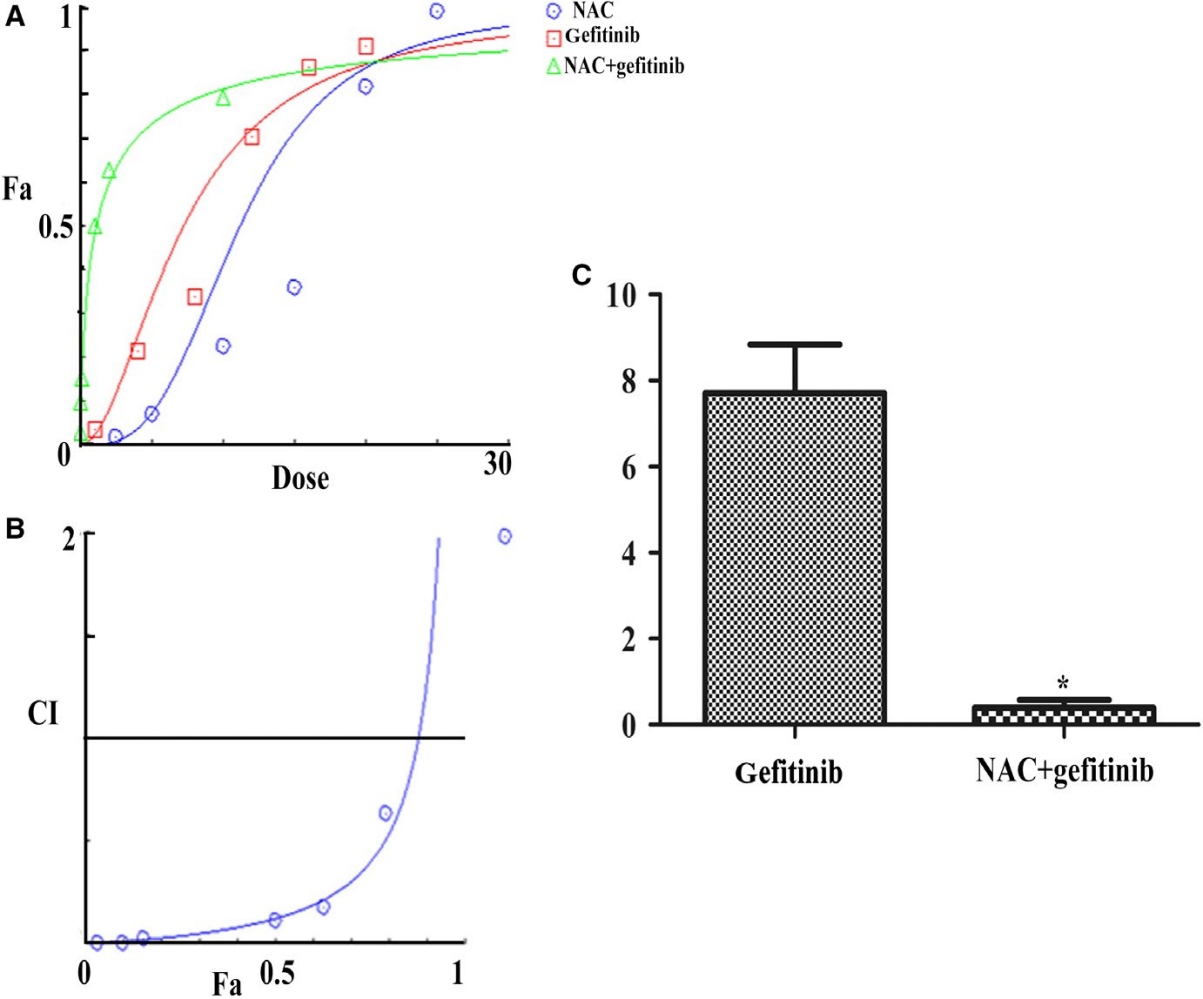
CalcuSyn‐based analysis of the N‐acetylcysteine (NAC) and gefitinib combination. A, Analysis of synergistic effect between NAC and gefiinib. B, CI values at different levels of growth inhibition effect. C, IC50 of gefitinib in alone group and combination group. *P < .001 between NAC + gefitinib and gefitinib; Fa: fraction affected; IC50: half maximal inhibitory concentration; CI: combination index

### 3.2 CalcuSyn‐based analysis of NAC and gefitinib combination treatment

The constant combination ratio experiments were carried out at an equipotency ratio approximating their individual IC50 (IC50_NAC_: IC50_gefitinib_ ≈ 2:1), which made sure the effect of each drug in combination was roughly equal. Figure 1A showed the dose‐response curves for PC‐9/GR cells exposed to NAC, gefitinib and both. CI values of the group treated with a combination of both drugs in different fractional cell growth inhibition (Fa) were shown in Figure 1B. CI values of less than 1 were acquired from the combination group, demonstrating that the two drugs must have a synergistic effect on growth inhibition. Then, PC‐9/GR cells were treated with 5 mmol/L of NAC adding different concentrations of gefitinib. We found that the IC50 of gefitinib was 0.3986μmol/L in the combination group, which was lower than gefitinib alone (*P* < .001; Figure 1C).

### 3.3 Combination of NAC and gefitinib inhibited migration and invasion of PC‐9/GR cells

**Figure 2:**
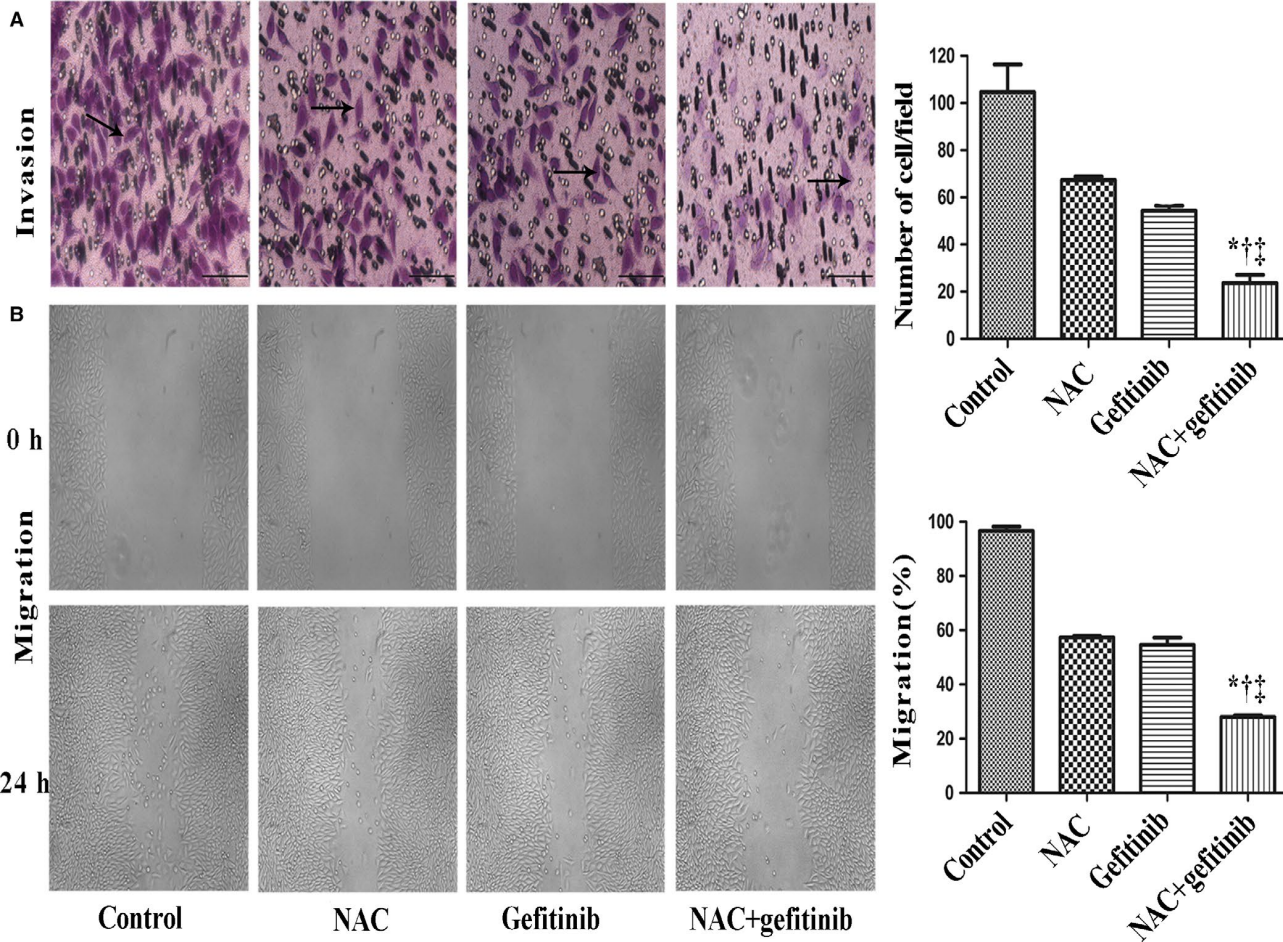
CalcuSyn‐based analysis of the N‐acetylcysteine (NAC) and gefitinib combination. A, Cells were pretreated with NAC or gefitinib alone and combination, PC‐9/GR cells passed through the matriged was lower than other groups. B, Wound healing assays showed that the distance of cell migration in different group for 0 h, 24 h. NAC + gefitinib vs control, *P < .001; NAC + gefitinib vs NAC, ^†^ *P* < .01; NAC + gefitinib vs gefitinib, ^‡^ *P* < .01. Scale bars: 100 µm. NAC, 5 mmol/L; gefitinib, 2 μmol/L

To investigate whether NAC (5 mmol/L) in combination with gefitinib (2 μmol/L) had an impact on biological behavior of PC‐9/GR cells, we performed migration and invasion assays. After 48 hours of treatment with NAC or gefitinib alone or in combination (NAC + gefitinib group), the number of cells passing through the Matrigel decreased in the NAC + gefitinib group compared to that in either alone group (Figure 2A). Cell migration assay showed that the distance of cell migration was the shortest in the NAC + gefitinib group (Figure 2B). These data illustrated that NAC in combination with gefitinib could inhibit the invasion and migration of PC‐9/GR cells.

### 3.4 NAC in combination with gefitinib promoted apoptotic rate in PC‐9/GR cells

**Figure 3:**
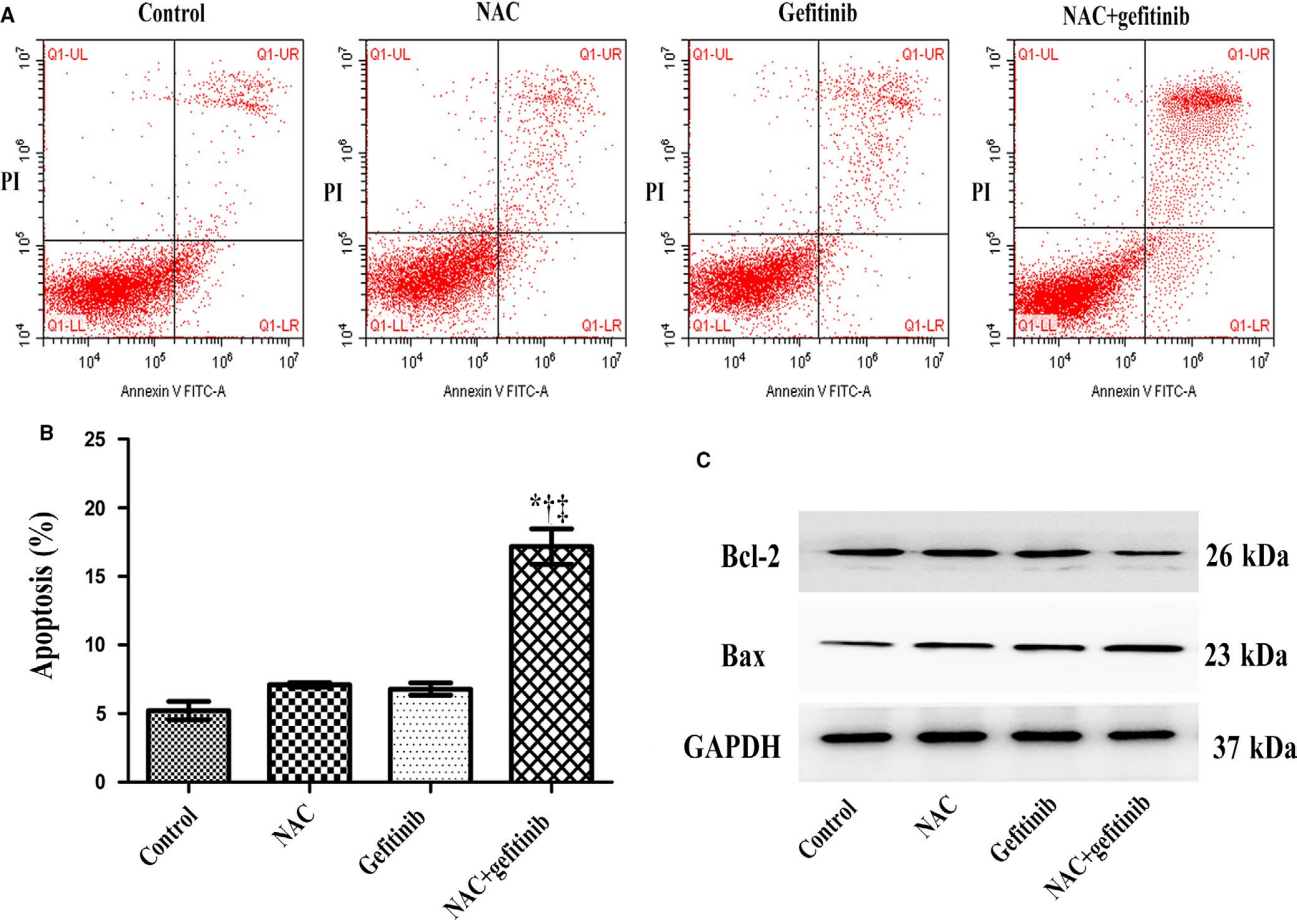
N‐acetylcysteine (NAC) in combination with gefitinib induced PC‐9/GR cells apoptosis. A and B, NAC combination with gefitinib induced apoptotic. C, The level of Bcl‐2 protein expression was low, while Bax was high expression. NAC + gefitinib vs control, *P < .001; NAC + gefitinib vs NAC, ^†^ *P* < .01; NAC + gefitinib vs gefitinib, ^‡^ *P* < .01

Furthermore, we detected the apoptosis of PC‐9/GR cells under different treatments using flow cytometry analysis. NAC + gefitinib caused more apoptotic cells compared with NAC or gefitinib alone did (*P* < .01; Figure 3A,B). Bax and Bcl‐2 are known as pro‐apoptotic and anti‐apoptotic molecules, respectively. The protein level of Bcl‐2 was decreased in the NAC + gefitinib group. While treatment with NAC or geftinib alone led to higher expression of Bax (Figure 3C). The trend of protein level was observed for Bcl‐2 and Bax in the combination group compared to the other groups. These results demonstrated that NAC in combination with gefitinib promoted apoptosis of PC‐9/GR cells.

### 3.5 NAC in combination with gefitinib reversed EMT and inhibited Src activation in PC‐9/GR cells

**Figure 4:**
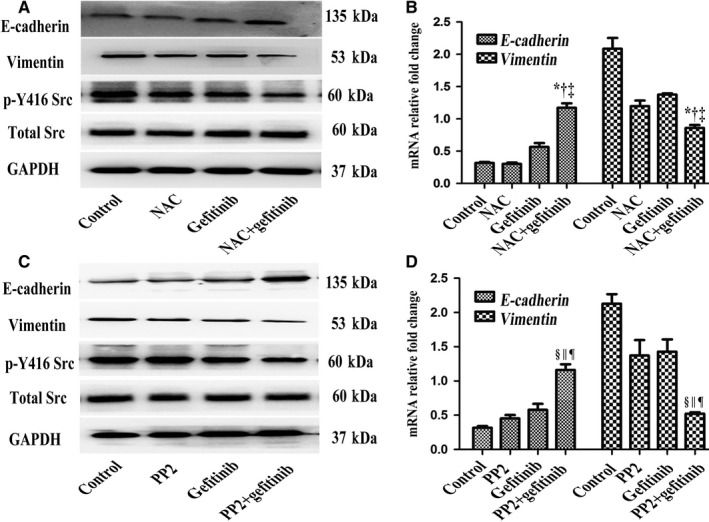
N‐acetylcysteine (NAC) in combination with gefitinib reversed EMT and inhibited Src activation in PC‐9/GR cells. A and B, NAC combination with gefitinib could inhibited Src activiation and reversed EMT. C and D, PP2 combination with gefitinib could inhibite Src activation and reverse EMT in PC‐9/GR cells. NAC + gefitinib vs control, *P < .001; NAC + gefitinib vs NAC, ^†^ *P* < .01; NAC + gefitinib vs gefitinib, ^‡^ *P* < .01; PP2 + gefitinib vs control, ^§^ *P < *.001; PP2 + gefitinib vs NAC, ^||^ *P* < .01; PP2 + gefitinib vs gefitinib, ^¶^ *P* < .01. PP2, 10 µmol/L

We next explored the underlying mechanism that led to the superior efficacy of NAC in combination with gefitinib. Western blot analysis showed that NAC in combination with gefitnib facilitated E‐cadherin expression and inhibited Vimentin expression in PC‐9/GR cells. In addition, the protein level of p‐Src was decreased in the combination group (Figure 4A). In qRT‐PCR analyses, expression of the E‐cadherin increased and vimentin was decreased in the NAC + gefitinib group (Figure 4B). Then, we treated PC9/GR with PP2 (a potent inhibitor of Src, 10 µmol/L) in combination with gefitinib, which displayed the similar results as NAC + gefitinib did (Figure 4C,D). These results indicated that NAC in combination with gefitinib could inhibit Src activation and reverse EMT.

## 4 DISCUSSION

NSCLC patients with EGFR mutations initially get good responses to EGFR‐TKIs. However, these patients gradually acquire drug resistance inevitably. Therefore, it is of necessity to develop novel strategies to delay or overcome the acquired resistance to EGFR‐TKIs. One of the interesting approaches would be the combination treatment with alternative drug in addition to gefitinib. Drug combination is widely used and has become the primary treatment modality for cancer.^26^ A combination of gefitinib with metformin shows a synergistic effect and increases sensitivity of patients with lung cancer to gefitinib.^27^ In this study, we used NAC in combination with gefitinib, which could effectively overcome drug resistance to gefitinb.

NAC is a precursor of glutathione, a powerful antioxidant used in anti‐tumor research. It has been reported that NAC inhibits proliferation and invasive behavior of human cancer cells in vitro, including colorectal cancer, bladder cancer, prostate cancer, tongue cancer, and lung carcinoma.^28^, ^29^, ^30^, ^31^ Our previous study showed that NAC could overcome gefitinib resistance induced by cigarette smoke extract. NAC has been proven to inhibit the growth of lung carcinomas by reducing cell proliferation and facilitating apoptosis in tobacco carcinogen‐treated A/J mice.^32^ NAC exerts inhibitory effect on tumor growth via modulation of EGFR/AKT signaling and HBP1 expression in EGFR‐overexpressed oral cancer.^33^ It can be inferred that NAC has promising potential to be a novel anticancer agent. In this study, we demonstrated that NAC could overcome gefitinib resistance by inhibiting Src activation and reversing EMT in PC‐9/GR cells when used in combination with gefitinib. It has been demonstrated that Src is a key regulator of EMT in cancer cells.^34^, ^35^ Our research verified that NAC modulated EMT of lung cancer cells by inhibiting the activation of Src, which might clarify the underlying mechanism theoretically.

EMT is one of hallmarks in cancer and plays a crucial role in the development and progression of most solid tumors.^36^ As a candidate mechanism of drug resistance, EMT plays a crucial role in acquired EGFR‐TKIs resistance as reported by different research groups.^37^, ^38^ It has been demonstrated that EMT could be regarded as a predictor of therapy response in patients with NSCLC. Furthermore, there is a direct connection between EMT and EGFR‐TKIs sensitivity. Research has shown that E‐cadherin potentiates the sensitivity to gefitinb to improve the effect.^39^ Notably, EMT co‐occurs with EGFR T790M mutations in a study using re‐biopsies.^40^ Multiple signaling networks modulate EMT, such as transforming growth factor‐β1 (TGF‐β1), mitogen‐activated protein (MAP) kinases, RHOA, AKT, and STAT3. Multiple molecules are involved in EMT, including Src, a key oncogene.

Src is the first oncogene ever identified, and its family members have also been recognized as potential targets in cancer therapy. Activation of Src promotes numerous pathological processes, including invasion, migration, proliferation, and angiogenesis in a variety of cancers.^41^ Increased Src activity boosts EMT process, while Src inhibition suppresses this process. Moreover Src‐mediated EMT is involved in the chemotherapy resistance of cancers.^42^, ^43^ Some studies concentrate on the combination of EGFR inhibitors and Src inhibitors. A previous study has revealed that the efficacy of Src inhibitors combined with EGFR inhibitors is synergistic, and Src inhibitors could improve gefitinib resistance in NSCLC with EMT.^44^ Our previous studies have shown that Src is involved in cigarette smoke‐induced EMT and EGFR‐TKI resistance, and Src inhibition sensitizes resistant cells to gefitinib. However, further investigations are needed to determine whether this combination in vivo could delay drug resistance and prolong patient progression‐free survival (PFS) and overall survival (OS).

A lack of in vivo study is an obvious limitation in the current study, and we will conduct in vivo experiments in animal models to verify the cytological results in this study. We have not further investigated the up‐and‐down pathway of apoptotic proteins, which is another limitation of this study. To prove the synergistic effect of this combination therapy for EGFR mutant lung cancer, the future study should demonstrate with other EGFR mutant lung cancer cell lines. In addition to gefitinib, osimertinib has obtained positive results from the phase 3 AURA trial and phase 3 FLAURA trial.^45^, ^46^ With the approval of osimertinib in Nov 2015 (https://www.fda.gov/), it will be clinically meaningful if osimertinib has the similar effect when combined with NAC.

In conclusion, we used the combination index value to evaluate the efficacy of NAC and gefitinib combination treatment. Our results demonstrated that NAC had synergistic effect with gefitinib. These two drugs used in combination overcame the resistance of PC‐9/GR to gefitinib by inhibiting Src activation and reversing EMT. Thus, these findings highlighted a novel insight into overcoming gefitinib resistance and provided a potential strategy for NSCLC patients.

## CONFLICT OF INTEREST

We declare no conflicts of interest in association with this study.

## References

[1] Bray F , Ferlay J , Soerjomataram I , Siegel RL , Torre LA , Jemal A . Global cancer statistics 2018: GLOBOCAN estimates of incidence and mortality worldwide for 36 cancers in 185 countries. CA Cancer J Clin. 2018;68:394‐424. 30207593 10.3322/caac.21492

[2] Siegel RL , Miller KD , Jemal A . Cancer statistics, 2019. CA Cancer J Clin. 2019;69:7‐34. 30620402 10.3322/caac.21551

[3] Pao W , Chmielecki J . Rational, biologically based treatment of EGFR‐mutant non‐small‐cell lung cancer. Nat Rev Cancer. 2010;10:760‐774. 20966921 10.1038/nrc2947 PMC3072803

[4] Nguyen KS , Neal JW . First‐line treatment of EGFR‐mutant non‐small‐cell lung cancer: the role of erlotinib and other tyrosine kinase inhibitors. Biologics. 2012;6:337‐345. 23055691 10.2147/BTT.S26558 PMC3459550

[5] Lee CK , Brown C , Gralla RJ , et al. Impact of EGFR inhibitor in non‐small cell lung cancer on progression‐free and overall survival: a meta‐analysis. J Natl Cancer Inst. 2013;105:595‐605. 23594426 10.1093/jnci/djt072

[6] Antonicelli A , Cafarotti S , Indini A , et al. EGFR‐targeted therapy for non‐small cell lung cancer: focus on EGFR oncogenic mutation. Int J Med Sci. 2013;10:320‐330. 23423768 10.7150/ijms.4609 PMC3575628

[7] Greenhalgh J , Dwan K , Boland A , et al. First‐line treatment of advanced epidermal growth factor receptor (EGFR) mutation positive non‐squamous non‐small cell lung cancer. Cochrane Database Syst Rev. 2016;CD010383. 27223332 10.1002/14651858.CD010383.pub2

[8] Jackman D , Pao W , Riely GJ , et al. Clinical definition of acquired resistance to epidermal growth factor receptor tyrosine kinase inhibitors in non‐small‐cell lung cancer. J Clin Oncol. 2010;28:357‐360. 19949011 10.1200/JCO.2009.24.7049 PMC3870288

[9] Jackman DM , Yeap BY , Sequist LV , et al. Exon 19 deletion mutations of epidermal growth factor receptor are associated with prolonged survival in non‐small cell lung cancer patients treated with gefitinib or erlotinib. Clin Cancer Res. 2006;12:3908‐3914. 16818686 10.1158/1078-0432.CCR-06-0462

[10] Riely GJ , Pao W , Pham D , et al. Clinical course of patients with non‐small cell lung cancer and epidermal growth factor receptor exon 19 and exon 21 mutations treated with gefitinib or erlotinib. Clin Cancer Res. 2006;12:839‐844. 16467097 10.1158/1078-0432.CCR-05-1846

[11] Zhang K , Yuan Q . Current mechanism of acquired resistance to epidermal growth factor receptor‐tyrosine kinase inhibitors and updated therapy strategies in human nonsmall cell lung cancer. J Cancer Res Ther. 2016;12:C131‐C137. 28230005 10.4103/0973-1482.200613

[12] Remon J , Moran T , Majem M , et al. Acquired resistance to epidermal growth factor receptor tyrosine kinase inhibitors in EGFR‐mutant non‐small cell lung cancer: a new era begins. Cancer Treat Rev. 2014;40:93‐101. 23829935 10.1016/j.ctrv.2013.06.002

[13] Tan CS , Gilligan D , Pacey S . Treatment approaches for EGFR‐inhibitor‐resistant patients with non‐small‐cell lung cancer. Lancet Oncol. 2015;16:e447‐e459. 26370354 10.1016/S1470-2045(15)00246-6

[14] Lin Y , Wang X , Jin H . EGFR‐TKI resistance in NSCLC patients: mechanisms and strategies. Am J Cancer Res. 2014;4:411‐435. 25232485 PMC4163608

[15] Nieto MA , Huang RY , Jackson RA , Thiery J . Emt: 2016. Cell. 2016;166(1):21‐45. 27368099 10.1016/j.cell.2016.06.028

[16] Bottoni P , Isgro MA , Scatena R . The epithelial‐mesenchymal transition in cancer: a potential critical topic for translational proteomic research. Expert Rev Proteomics. 2016;13:115‐133. 26567562 10.1586/14789450.2016.1112742

[17] Ye M , Zhang Y , Gao H , et al. Activation of the aryl hydrocarbon receptor leads to resistance to EGFR TKIs in non‐small cell lung cancer by activating Src‐mediated bypass signaling. Clin Cancer Res. 2018;24:1227‐1239. 29229632 10.1158/1078-0432.CCR-17-0396

[18] Ye M , Zhang Y , Zhang X , et al. Targeting FBW7 as a strategy to overcome resistance to targeted therapy in non‐small cell lung cancer. Cancer Res. 2017;77:3527‐3539. 28522751 10.1158/0008-5472.CAN-16-3470

[19] Song J , Zhong R , Huang H , et al. Combined treatment with Epimedium koreanum Nakai extract and gefitinib overcomes drug resistance caused by T790M mutation in non‐small cell lung cancer cells. Nutr Cancer. 2014;66:682‐689. 24738693 10.1080/01635581.2014.895392

[20] Zhan Y , Chen Y , Liu R , Zhang H , Zhang Y . Potentiation of paclitaxel activity by curcumin in human breast cancer cell by modulating apoptosis and inhibiting EGFR signaling. Arch Pharm Res. 2014;37:1086‐1095. 24318305 10.1007/s12272-013-0311-3

[21] Matthews H , Deakin J , Rajab M , Idris‐Usman M , Nirmalan NJ . Investigating antimalarial drug interactions of emetine dihydrochloride hydrate using CalcuSyn‐based interactivity calculations. PLoS ONE. 2017;12:e0173303. 28257497 10.1371/journal.pone.0173303 PMC5336292

[22] Chou TC . Theoretical basis, experimental design, and computerized simulation of synergism and antagonism in drug combination studies. Pharmacol Rev. 2006;58:621‐681. 16968952 10.1124/pr.58.3.10

[23] Chou TC , Talalay P . Quantitative analysis of dose‐effect relationships: the combined effects of multiple drugs or enzyme inhibitors. Adv Enzyme Regul. 1984;22:27‐55. 6382953 10.1016/0065-2571(84)90007-4

[24] Zhang L , Li J , Hu J , et al. Cigarette smoke extract induces EGFR‐TKI resistance via promoting EGFR signaling pathway and ROS generation in NSCLC cell lines. Lung Cancer. 2017;109:109‐116. 28577939 10.1016/j.lungcan.2017.05.011

[25] Li D , Zhang L , Zhou J , Chen H . Cigarette smoke extract exposure induces EGFR‐TKI resistance in EGFR‐mutated NSCLC via mediating Src activation and EMT. Lung Cancer. 2016;93:35‐42. 26898612 10.1016/j.lungcan.2015.12.007

[26] Zhang N , Fu JN , Chou TC . Synergistic combination of microtubule targeting anticancer fludelone with cytoprotective panaxytriol derived from panax ginseng against MX‐1 cells in vitro: experimental design and data analysis using the combination index method. Am J Cancer Res. 2016;6:97‐104. 27073727 PMC4759401

[27] Li L , Han R , Xiao H , et al. Metformin sensitizes EGFR‐TKI‐resistant human lung cancer cells in vitro and in vivo through inhibition of IL‐6 signaling and EMT reversal. Clin Cancer Res. 2014;20:2714‐2726. 24644001 10.1158/1078-0432.CCR-13-2613

[28] Piskounova E , Agathocleous M , Murphy MM , et al. Oxidative stress inhibits distant metastasis by human melanoma cells. Nature. 2015;527:186‐191. 26466563 10.1038/nature15726 PMC4644103

[29] Amini A , Masoumi‐Moghaddam S , Ehteda A , Morris DL . Bromelain and N‐acetylcysteine inhibit proliferation and survival of gastrointestinal cancer cells in vitro: significance of combination therapy. J Exp Clin Cancer Res. 2014;33:92. 25425315 10.1186/s13046-014-0092-7 PMC4245783

[30] Hann SS , Zheng F , Zhao S . Targeting 3‐phosphoinositide‐dependent protein kinase 1 by N‐acetyl‐cysteine through activation of peroxisome proliferators activated receptor alpha in human lung cancer cells, the role of p53 and p65. J Exp Clin Cancer Res. 2013;32:43. 23867003 10.1186/1756-9966-32-43 PMC3720217

[31] Lee YJ , Lee DM , Lee CH , et al. Suppression of human prostate cancer PC‐3 cell growth by N‐acetylcysteine involves over‐expression of Cyr61. Toxicol In Vitro. 2011;25:199‐205. 21055460 10.1016/j.tiv.2010.10.020

[32] Conaway CC , Wang CX , Pittman B , et al. Phenethyl isothiocyanate and sulforaphane and their N‐acetylcysteine conjugates inhibit malignant progression of lung adenomas induced by tobacco carcinogens in A/J mice. Cancer Res. 2005;65:8548‐8557. 16166336 10.1158/0008-5472.CAN-05-0237

[33] Lee MF , Chan CY , Hung HC , Chou IT , Yee AS , Huang CY . N‐acetylcysteine (NAC) inhibits cell growth by mediating the EGFR/Akt/HMG box‐containing protein 1 (HBP1) signaling pathway in invasive oral cancer. Oral Oncol. 2013;49:129‐135. 22944050 10.1016/j.oraloncology.2012.08.003

[34] Fang D , Chen H , Zhu JY , et al. Epithelial‐mesenchymal transition of ovarian cancer cells is sustained by Rac1 through simultaneous activation of MEK1/2 and Src signaling pathways. Oncogene. 2017;36:1546‐1558. 27617576 10.1038/onc.2016.323 PMC5346482

[35] Srivastava K , Pickard A , Craig SG , et al. DeltaNp63gamma/SRC/Slug signaling axis promotes epithelial‐to‐mesenchymal transition in squamous cancers. Clin Cancer Res. 2018;24:3917‐3927. 29739791 10.1158/1078-0432.CCR-17-3775 PMC6098695

[36] Hanahan D , Weinberg RA . Hallmarks of cancer: the next generation. Cell. 2011;144:646‐674. 21376230 10.1016/j.cell.2011.02.013

[37] Sesumi Y , Suda K , Mizuuchi H , et al. Effect of dasatinib on EMT‐mediated‐mechanism of resistance against EGFR inhibitors in lung cancer cells. Lung Cancer. 2017;104:85‐90. 28213007 10.1016/j.lungcan.2016.12.012

[38] Sato H , Shien K , Tomida S , et al. Targeting the miR‐200c/LIN28B axis in acquired EGFR‐TKI resistance non‐small cell lung cancer cells harboring EMT features. Sci Rep. 2017;7:40847. 28084458 10.1038/srep40847 PMC5233972

[39] Witta SE , Gemmill RM , Hirsch FR , et al. Restoring E‐cadherin expression increases sensitivity to epidermal growth factor receptor inhibitors in lung cancer cell lines. Cancer Res. 2006;66:944‐950. 16424029 10.1158/0008-5472.CAN-05-1988

[40] Uramoto H , Shimokawa H , Hanagiri T , Kuwano M , Ono M . Expression of selected gene for acquired drug resistance to EGFR‐TKI in lung adenocarcinoma. Lung Cancer. 2011;73:361‐365. 21315472 10.1016/j.lungcan.2011.01.008

[41] Dehm SM , Bonham K . SRC gene expression in human cancer: the role of transcriptional activation. Biochem Cell Biol. 2004;82:263‐274. 15060621 10.1139/o03-077

[42] Nagathihalli NS , Merchant NB . Src‐mediated regulation of E‐cadherin and EMT in pancreatic cancer. Front Biosci (Landmark Ed). 2012;17:2059‐2069. 22652764 10.2741/4037

[43] Wilson C , Nicholes K , Bustos D , et al. Overcoming EMT‐associated resistance to anti‐cancer drugs via Src/FAK pathway inhibition. Oncotarget. 2014;5:7328‐7341. 25193862 10.18632/oncotarget.2397 PMC4202126

[44] Yoshida T , Okamoto I , Okamoto W , et al. Effects of Src inhibitors on cell growth and epidermal growth factor receptor and MET signaling in gefitinib‐resistant non‐small cell lung cancer cells with acquired MET amplification. Cancer Sci. 2010;101:167‐172. 19804422 10.1111/j.1349-7006.2009.01368.x PMC11158912

[45] Mok TS , Wu YL , Ahn MJ , et al. Osimertinib or platinum‐pemetrexed in EGFR T790M‐positive lung cancer. N Engl J Med. 2017;376:629‐640. 27959700 10.1056/NEJMoa1612674 PMC6762027

[46] Soria JC , Ohe Y , Vansteenkiste J , et al. Osimertinib in untreated EGFR‐mutated advanced non‐small‐cell lung cancer. N Engl J Med. 2018;378:113‐125. 29151359 10.1056/NEJMoa1713137

