# Supplementary material for: Combined treatment with N‐acetylcysteine and gefitinib overcomes drug resistance to gefitinib in NSCLC cell line
Source: Cancer Med. 2019 Dec 31;9(4):1495–502. doi: 10.1002/cam4.2610 (PMC7013061; doi:10.1002/cam4.2610)
Supplement: Supplementary file 1 [file CAM4-9-1495-s001.docx]

**
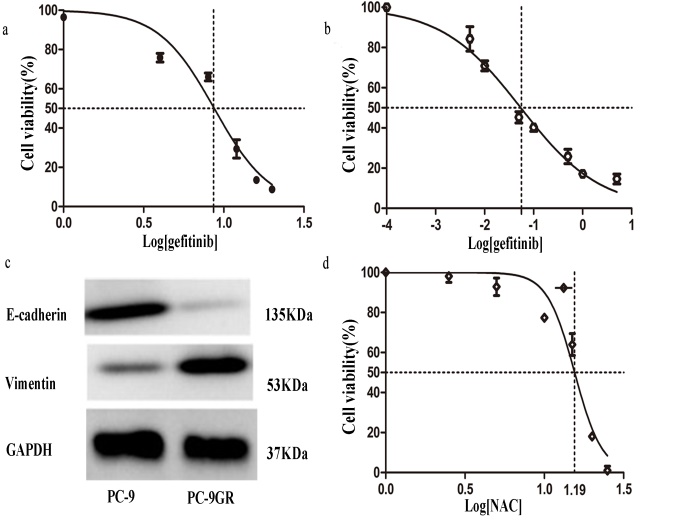
**

**Supplementary FIGURE 1** Determination of dose-response curves and PC-9/GR EMT phenotype characteristics. (a) Dose-response curves of gefitinib to PC-9/GR cells. (b) Dose-response curves of gefitinib to PC-9 cells. (c) Western blot analysis of changes of EMT phenotype characteristics in both PC-9 and PC-9/GR cells. (d) Dose-response curves of NAC to PC-9/GR cells. PC-9 (gefitinib-sensitive cells); PC-9/GR (gefitinib-resistant cells); NAC (N-acetylcysteine).
